# An Importance of Long-Term Clinical Analysis to Accurately Diagnose Calves Persistently and Acutely Infected by Bovine Viral Diarrhea Virus 2

**DOI:** 10.3390/v13122431

**Published:** 2021-12-03

**Authors:** Yusuke Goto, Gakuji Yaegashi, Kazuhiro Fukunari, Tohru Suzuki

**Affiliations:** 1 Central Iwate Prefectural Livestock Health and Hygiene Center, Takizawa 020-0605, Iwate, Japan; y.gotou@pref.iwate.jp (Y.G.); g-yaegashi@pref.iwate.jp (G.Y.); fukunari@pref.iwate.jp (K.F.); 2 Division of Zoonosis Research, Sapporo Research Station, National Institute of Animal Health, NARO, Sapporo 062-0045, Hokkaido, Japan; 3 Division of Hygiene Management, Sapporo Research Station, National Institute of Animal Health, NARO, Sapporo 062-0045, Hokkaido, Japan

**Keywords:** bovine viral diarrhea virus, acutely infected, persistently infected, calf

## Abstract

Bovine viral diarrhea virus (BVDV) infection results in a wide variety of clinical manifestations and is a pathogen that is able to cause huge economic losses in the cattle industry worldwide. It is important to identify cattle that are persistently infected (PI) by BVDV within the herd as early as possible because PI animals are the main reservoir of the virus. In contrast, cattle who are acutely infected (AI) with BVDV show various clinical signs, but most cattle show either mild symptoms or are asymptomatic. In general, AI and PI animals can be distinguished by repeat testing within an interval of at least 21 days. However, we found a rare case of a BVDV2-infected AI animal with long-term viral presence, making it indistinguishable from PI through two tests within an interval of 21 days. As a result, we diagnosed one infected animal as AI after 35 days from the initial sample collection via multiple analyses. Our findings recommend performing an additional test using samples that have been collected after 14–21 days from the second sample collection in cases where it is difficult to accurately differentiate an AI diagnosis from a PI diagnosis after only two tests. Additionally, our analysis exhibits that monitoring the number of copies of viruses with similar genomes in the sera by means of quantitative real-time RT-PCR through several sample collections periods might be useful to distinguish AI from PI. Furthermore, our data suggest that the AI animals with a long-term viral presence who show test results similar to those of PI animals might be the result of a coincidental combination of various factors that are present in cattle fields. These findings provide useful information that can be used to improve the diagnosis of BVDV in the field.

## 1. Introduction

Bovine Pestiviruses, also known as bovine viral diarrhea viruses (BVDVs), belong to the genus *Pestivirus* of the family *Flaviviridae* and are one of important causative pathogens among cattle disorders and that have a worldwide economic impact [1,2,3,4]. BVDV is a positive single-stranded RNA virus that is currently divided into Pestivirus A (BVDV1) and B (BVDV2), according to genetic and antigenic characteristics [5,6,7]. Additionally, BVDVs are also divided into two biotypes, non-cytopathic (ncp) and cytopathic (cp), with this division corresponding to its cytopathic effect on cells [8]. Only ncp-type BVDV is able to produce persistently infected (PI) calves, and it may result in the development mucosal disease [9,10,11]. On the other hand, cp-type BVDV has been only isolated from animals with mucosal disease [12,13,14]. Furthermore, there are some reports indicating the existence of highly pathogenic BVDV2 in several countries, but this has not been found to be true in Japan [15,16,17].

Cattle who are acutely infected (AI) with BVDV sometimes show various clinical signs such as slight fever, leukopenia, respiratory disorders, diarrhea, and abortion; however, AI animals seem to be mild or asymptomatic in most cases [18,19,20]. In addition, the AI animals that are seronegative to BVDV exhibit a short-term viremia and virus shedding in nasal and in other forms of discharge [21,22]. Furthermore, AI animals show seroconversion within two weeks after BVDV infection and recover within three weeks after BVDV infection [23,24,25]. On the other hand, PI animals show no seroconversion and continuous virus shedding [26]. Therefore, the OIE Terrestrial Manual recommends performing tests using two blood samples that have been collected within at least 21 days from the initial sample collection in order to distinguish AI and PI cases [27].

In this study, we found a field case of AI with a long-term presence of BVDV2 that was indistinguishable from a PI case, even after two tests were conducted within 21 days of each other. Therefore, we attempted to use this case in order to obtain valuable information that would help to make it possible to distinguish AI and PI cases through the use of multiple, with multiple tests using various analyses being conducted for this field case.

## 2. Materials and Methods

### 2.1. Cattle Farms

We investigated three (nos. A1–A3) and four (nos. B1–B4) calves who had no clinical signs of BVDV from two dairy farms, dairy farm A and B, respectively. On calf (no. A3) presented with diarrhea. Farm A maintains approximately 150 cows, 30 heifers, and 20 calves, and farm B maintains approximately 60 cows, 20 heifers, and 15 calves.

At farm A, the newborn calves were moved to the calf barn within one day of being born and were in individual pens until they were four weeks old. At farm A, the calves were able to interact with the calves in the neighbouring pens. Two calves (nos. A1 and A3) were in the pens next to each other, and one calf (no. A2) was also in a nearby pen during this time. After four weeks, they were moved to the breeding group; each group comprised about 20 animals. The calves could move freely within the fenced area.

At farm B, the newborn calves were moved to the calf barn within one day of being born and were kept in individual pens until they were four months old. The calves were able to interact with the cows in the neighbouring pens. Two calves (nos. B1 and B2) were in pens that were close to each other, but two calves (nos. B3 and B4) were in pens that were a little farther away. After four months, they shifted to the breeding group; each group comprised 10 animals. The calves could move freely within the fenced area.

Vaccines containing BVDV were not used on either farm. In addition, the calves were fed the colostrum that had been previously collected and cryopreserved from other dams.

### 2.2. Sampling

All serum samples were taken for routine PI cattle detection screening. A detailed sample collection schedule is shown in Table 1. Sera were collected two or three times from the seven study calves at interval that were approximately 21 days long from September 2017 to January 2018. Additionally, serum samples were collected from 143 milking cows (24 to 106 months old) and 53 milking cows (24 to 119 months old) who were maintained at farm A and B, respectively, in order to investigate neutralizing antibody titers against BVDVs. All of the samples were collected as a part of routine diagnostic procedures; hence, permission concerning animal ethics was not required.

### 2.3. RNA Extraction and Real-Time RT-PCR for the Detection of Bovine Viral Diarrhea Virus

Viral RNA was extracted from the sera using a QIAamp Viral RNA Mini Kit according to the manufacturer’s instructions (Qiagen, Hilden, Germany). The VetMAX-Gold BVDV PI Detection Kit (Thermo Fisher Scientific, Waltham, MA, USA) was used for real-time RT-PCR (RT-qPCR). The reaction volume of 25 μL was composed of 12.5 μL 2× RT-PCR buffer, 1 μL 25× BVDV primers and probe mixture, 1 μL 25× RT-PCR enzyme mix, 2.5 μL nuclease free water, and 8 μL sample RNA. RT-qPCR was performed using serial 10-fold dilutes of standard BVDV RNA (10,000 copies/μL) in addition to the sera samples with Applied Biosystems 7500 Real-Time PCR systems (Thermo Fisher Scientific, Carlsbad, CA, USA) under the following conditions: 45 °C for 10 min and 95 °C for 10 min followed by 40 cycles of 95 °C for 15 s and 60 °C for 45 s. The results obtained from RT-qPCR were analyzed using ABI 7500 software version 2.0 and the genomic equivalent (GE) copies of each sample were estimated based on the standard curves in addition to the Ct values. Based on the manufacturer’s instructions, samples with Ct values <38 and ≦31 were positive for BVDV and putative PI animals, respectively.

### 2.4. Virus Isolation and the Immunoperoxidase Monolayer Assay

Virus isolation from sera was performed according to the immunoperoxidase monolayer assay as previously reported [28]. Bovine fetal muscular (BFM: originally produced in our laboratory) cells were maintained under the following conditions: EMEM supplemented with 3 mg/mL tryptose phosphate broth (Becton Dickinson, San Jose, CA, USA), 0.292 mg/mL L-glutamine (FUJIFILM Wako Pure Chemical Corporation, Osaka, Japan), 1.125 mg/mL sodium hydrogen carbonate (FUJIFILM Wako Pure Chemical Corporation, Osaka, Japan), and 5% BVDV antigen- and antibody-free bovine serum (Japan Bio Serum, Hiroshima, Japan). Serial twofold dilutions of sera were placed into four wells of 96-well plates with 0.1 mL of each dilution. Thereafter, BFM cells (approximately 1.5 × 10^4^ cells) were added into all of the wells. Plates were incubated at 37 °C in 5% CO_2_ for 4 days, air-dried after medium removal, and fixed at 80 °C for 1 h. Fixed cells were reacted with 50 μL mouse IgG anti-pestivirus monoclonal antibody, JCU/BVD/CF10 (TropBio Pty Ltd., Queensland, Sydney, Australia) and were diluted to 1:500 with phosphate-buffered saline (PBS) containing 1% bovine serum albumin (BSA) (FUJIFILM Wako Pure Chemical Corporation, Osaka, Japan) at 37 °C for 30 min. Thereafter, the cells were washed twice in PBS containing 0.1% Tween 20 (PBST) and were reacted with 50 μL horseradish peroxidase conjugated goat anti-mouse IgG (Bio-Rad Laboratories, Inc. Hercules, CA, USA) diluted to 1:1000 with PBS containing 1% BSA at 37 °C for 30 min. The cells were washed twice in PBST yet again, were reacted with a 100 μL solution containing a 5% 3-amino-9-ethylcarbazole (AEC) solution (20 mg AEC dissolved in 2.5 mL of dimethyl sulfoxide) and 0.015% H_2_O_2_ in 50 mM acetate buffer (pH 5.0), and were incubated in the dark at 37 °C for 30 min. A positive reaction was characterized by the appearance of reddish-brown staining in the cytoplasm and no staining in the nucleus.

### 2.5. Antigen Detection Enzyme-Linked Immunosorbent Assay (AgELISA)

Serum samples were tested via an antigen detection enzyme-linked immunosorbent assay (AgELISA) for BVDV using the IDEXX BVDV Ag/Serum Plus Test according to the manufacturer’s instructions (IDEXX Laboratories, Bern, Switzerland). AgELISA was designed for the detection of antigens against BVDV in bovine serum, plasma, and whole blood samples. A microtitration format has been configured by immobilizing specific monoclonal antibodies for BVDV (Erns) on the plate to capture BVDV antigen in the sample. The corrected OD value of the sample was calculated using a 450 nm absorbance that had been obtained from test samples (S) and by subtracting the absorbance of the negative control (N). Finally, the calculated OD value was the S-N value. Samples with S-N values ≦0.300 were considered negative for the BVDV antigen. Samples with S-N values >0.300 were considered positive.

### 2.6. Virus Neutralization Test

The virus neutralization test was performed according to a method that has been reported previously [29]. Each 50 μL of serum was serially diluted twofold with 50 μL EMEM supplemented with 3 mg/mL tryptose phosphate broth (Becton Dickinson, Sanjose, CA, USA), 0.292 mg/mL L-glutamine (FUJIFILM Wako Pure Chemical Corporation, Osaka, Japan), and 1.125 mg/mL sodium hydrogen carbonate (FUJIFILM Wako Pure Chemical Corporation) on 96-well plates. An equal volume (each 50 μL) of two representative BVDV strains, Nose (BVDV1) and KZ91-CP (BVDV2), each containing 200 TCID_50_/0.1 mL was added to each well and incubated at 37 °C for 1 h. Thereafter, 100 μL of BFM cells (approximately 1.5 × 10^4^ cells) was added into all of the wells and incubated at 37 °C in 5% CO_2_ for 5 days. The appearance of the cytopathogenic effect (CPE) was observed using a microscope (Olympus Corporation, Tokyo, Japan). The virus neutralization titer for each serum sample was expressed as the reciprocal of the highest dilution that inhibited CPE. An antibody titer of less than two was considered to be negative.

### 2.7. Genotyping of Bovine Viral Diarrhoea Virus by Multiplex Real-Time RT-PCR

TaqMan Fast Virus 1-Step Master Mix (Thermo Fisher Scientific, Carlsbad, CA, USA) was used for BVDV genotyping by multiplex real-time RT-PCR (multiplex RT-qPCR). Primers and probes were designed with reference to a previous report (Supplementary Table S1) [30]. The reaction volume of 20 μL was composed of 5 μL TaqMan Fast Virus 1-Step Master Mix, 1 μL 20× the three primers and the probe mixtures, 12 μL nuclease free water, and a 2 μL sample of RNA. Multiplex RT-qPCR was performed using Applied Biosystems 7500 Real-Time PCR systems under the following conditions: 50 °C for 5 min and 95 °C for 20 s followed by 40 cycles of 95 °C for 3 s and 60 °C for 34 s. The results that were obtained herein were analyzed using ABI 7500 software version 2.0.

### 2.8. Phylogenetic Analysis for 5′-Untranslated Regions and E2 Regions from Pestiviurs Strains

The partial genomes of the 5′-untranslated region (UTR) and the E2 region were amplified using the SuperScript III One-step RT-PCR System with Platinum Taq DNA Polymerase (Thermo Fisher Scientific, Carlsbad, CA, USA) and primer sets that were previously reported (Supplementary Table S2) [31,32]. The nucleotide sequences of the PCR products were determined using the BigDye Termina-tor v1.1 Cycles Sequencing Kit on an automated ABI Prism 3130XL Genetic Analyzer (Thermo Fisher Scientific, Carlsbad, CA, USA). Each genomic sequence from the five BVDVs determined herein was submitted to the DNA Data Bank of Japan; the sequences are retrievable from GenBank (5′-UTR: LC600230, LC620256, LC620257, LC620262, LC620264, and E2: LC649241–LC649245). Phylogenetic analysis using the data added to the genomic sequences of other BVDV strains that were already available in GenBank into the genomic sequences of the five BVDV strains was performed using the maximum-likelihood method and 1000 bootstrap replicates using MEGA7 software [33,34]. The nucleotide sequences of other BVDV strains that were used in the analysis of 5′-UTR were as follows: BVDV1 strains, NADL (GenBank accession number: AF039181), Nose (AB019670), Osloss (AY279526), BVDV2 strains, 890 (U18059), KZ91-CP (AB003619), and 1373 (AF145967). Moreover, this analysis also included three BVDV1 (IW280314, IW281332, IW290358) and one BVDV2 (IW290670) strains that had been detected from PI animals from the Iwate Prefecture between 2016 and 2017 (LC600229, LC620258, LC620259, LC620261). In contrast, the nucleotide sequences of the other BVDV strains that were used in the E2 analysis were as follows: BVDV1 strains, NADL (M31182), Nose (AB033752), Osloss (M96687), BVDV2 strains, 890 (U18059), KZ91-CP (AB105685), and 1373 (AF145967). Moreover, this analysis also included three BVDV1 (IW280314, IW281332, IW290358) and one BVDV2 (IW290670) strains that had been previously detected from PI animals from the Iwate Prefecture between 2016 and 2017 (LC649246–LC649249).

## 3. Results

### 3.1. Diagnosis of Seven Calves Based on a Series of Analyses

Upon initial sample collection, the Ct values (19.3–36.2: GE copies 69,000–1) that were positive for BVDV were detected in the sera from six of seven calves (nos. A1–A3 and B2–B4) by RT-qPCR (Table 1). In addition, ncp-type BVDVs were isolated in the sera from four calves (nos. A1, A3, B3, and B4). Moreover, the analysis of the AgELISA showed S-N values (0.333–3.698) that were positive for BVDV in the sera from seven calves. Furthermore, the sera from seven calves showed titers (negative to 32 and negative to 32) against BVDV1 and BVDV2 in the virus neutralization test, respectively.

During the the second sample collection period, which occurred approximately 21 days after the initial sample collection, sera were collected from seven calves. Ct values (19.2–28.1: GE copies 72,000–280) that were positive for BVDV were obtained in the sera from four calves (nos. A1, A3, B3, and B4). In addition, ncp-type BVDVs and cp-type BVDV were isolated in the sera from four calves (nos. A1, A3, B3, and B4) and in the serum from one calf (no. A3), respectively. Moreover, the analysis of the AgELISA results showed S-N values (3.276–3.702) that were positive for BVDV in the sera from four calves (nos. A1, A3, B3, and B4). Furthermore, the sera from seven calves showed titers (negative to 32 and negative to 1024) against BVDV1 and BVDV2 in the virus neutralization test, respectively. Of them, the sera from three calves (nos. A2, B1, and B2) showed relatively higher titers (512 to 1024) against BVDV2 than those from the remaining four calves.

During the third sample collection period, which took place approximately 35 days after the initial sample collection period, serum was collected from one calf (no. A1). A Ct value (35.2: GE copies 3) that was positive for BVDV was detected in the serum. In addition, no BVDV was isolated in the serum. Moreover, the analysis of the AgELISA results showed an S-N value (1.030) that was positive for BVDV in the serum. Furthermore, the serum showed titers (4 and 4) against BVDV1 and BVDV2 in the virus neutralization test, respectively.

In addition, the BVDVs that were detected in these animals were classified as belonging to BVDV2 via BVDV genotyping by multiplex RT-qPCR (Table 1).

Collectively, these findings showed that the three calves (nos. A3, B3, and B4) who obtained positive results in the RT-qPCR, virus isolation, AgELISA tests and no seroconversion through two tests occurring within 21 days of each other were determined to be PI animals with BVDV2. On the other hand, three calves (nos. A2, B1, and B2) obtained negative results in the RT-qPCR, virus isolation, and AgELISA tests and had undergone seroconversion through two tests occurring within 21 days of each other were identified as being AI animals with BVDV2. Regarding the last calf (no. A1), who demonstrated positive results for the RT-qPCR, virus isolation, AgELISA tests and no seroconversion at both the initial and second sample collection period was identified as a PI animal. However, the negative virus isolation and seroconversion results that were detected by virus neutralization test during the third sample collection period were mismatched to the PI animal criteria [27]. Consequently, these results suggested that the last calf (no. A1) was an AI animal with BVDV2.

### 3.2. Phylogenetic Analysis for 5′-Untranslated Regions and E2 Regions from Pestivirus Strains

**Figure 1:**
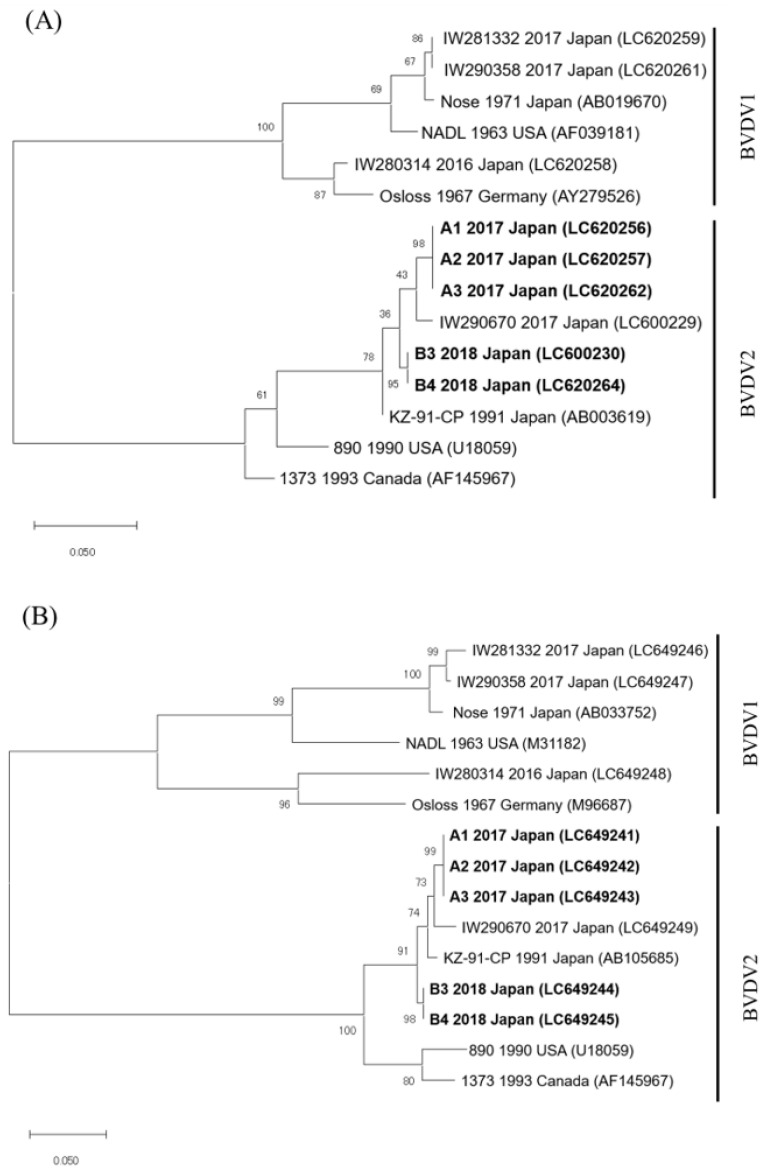
Phylogenetic tree using partial 5′-untranslated region (**A**) and partial E2 region (**B**) sequences from representative bovine viral diarrhea virus (BVDV) strains. The phylogenetic analysis was preformed using the nucleotide sequences of the 5′-UTR (product size: 245–248 base pairs) and the E2 region (product size: 657 base pairs) from representative BVDV strains, including the four BVDV strains (IW280314, IW281332, IW290358, and IW290670) isolated from cattle who were persistently infected in the Iwate Prefecture from 2016–2017. The tree was constructed using the maximum-likelihood method and 1000 bootstrap replicates on MEGA 7. Collection year, country, and GenBank accession strain number are shown. BVDV strains isolated from five calves in this study are shown in bold. Scale bar represents the number of substitutions per site.

The BVDVs that were detected from five (nos. A1–A3, B3, and B4) of seven calves were classified into BVDV2 the category via the phylogenetic analysis for the 5′-UTR and E2 genomic sequences (GenBank accession numbers, 5′-UTR: LC600230, LC620256, LC620257, LC620262, and LC620264, and E2: LC649241–LC649245) (Figure 1). The 5′-UTR and E2 genomic sequences of the BVDVs that were detected in three calves from farm A (nos. A1–A3) and two calves from farm B (nos. B3 and B4) were completely identical. Furthermore, the nucleotide sequence identities of the 5′-UTR and E2 sequences between farm A and farm B showed 97.6% (242/248 base pairs) and 97.9% (645/657 base pairs) similarity, respectively. In addition, the phylogenetic analyses for 5′-UTR and E2 revealed that these BVDVs were different from the highly pathogenic BVDV2 strains 890 and 1373.

### 3.3. Neutralizing Antibody Titers in Milking Cows

**Figure 2:**
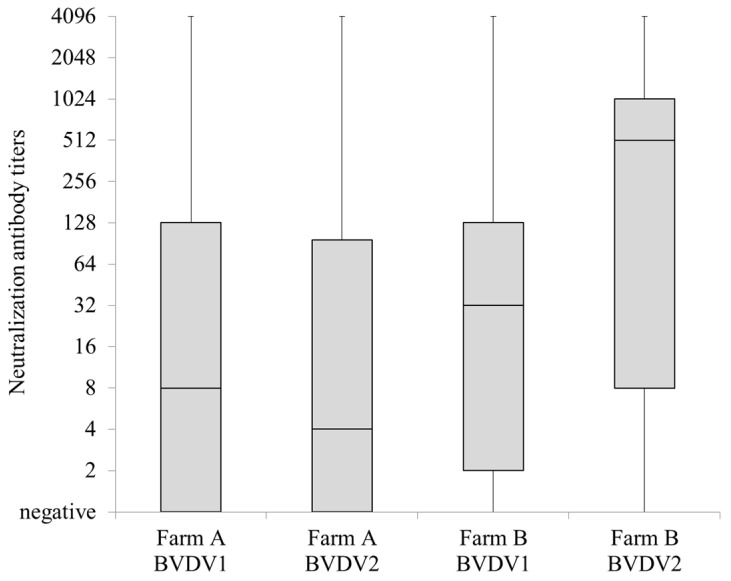
Summary of neutralizing antibody titers against bovine viral diarrhea virus 1 (BVDV1) and BVDV2 using sera from 143 milking cows and 53 milking cows maintained at farm A and B, respectively. Neutralization test was performed using sera serially diluted two-fold up to 4096 times at maximum. Negative was represented as 1.

Sera from 143 milking cows (24 to 106 ages in months) in the farm A and from 53 milking cows (24 to 119 ages in months) in the farm B showed median titers of 8 and 4 and 32 and 512 against BVDV1 and BVDV2 in the virus neutralization test, respectively (Figure 2).

## 4. Discussion

Our study identified a rare field case of an AI animal with the long-term presence of BVDV2 in serum, resulting in the animal being indistinguishable from the PI animals, even after two tests conducted 21 days apart. Eventually, our study demonstrated that 35 days and multiple sample collection periods were need before we could diagnose that one calf (no. A1) as an AI animal with BVDV2.

During the initial and second sample collection periods, all of analyses were suggested three calves (nos. A3, B3, and B4) were PI animals, as they were shown to have positive Ct values as demonstrated by RT-qPCR, ncp-type virus isolation, high S-N values, and no seroconversion of the BVDV2 antibody. In contrast, all of the analyses that were based on the two sample collection periods from three other caves calves (nos. A2, B1, and B2) indicated that they were AI not PI animals, as they were shown to have negative Ct values as demonstrated by RT-qPCR, no virus isolation, low S-N values, and seroconversion of the BVDV2 antibody. All of the analyses using paired samples that had been collected from the last one calf (no. A1) suggested that the calf was a PI animal, as it was shown to have positive Ct values as demonstrated by RT-qPCR, ncp-type virus isolation, high S-N values, and no seroconversion of the BVDV2 antibody. Specifically, the Ct value (28.1: GE copies 280) in the serum from the calf at second sample collection period was matched to the threshold value (Ct value ≦ 31) of that of a putative BVDV PI animal, but it was more increased from the Ct value (19.3: GE copies 69,000) that was found in the serum at the initial sample collection period. Our previous study suggested that it is important to conduct tests using various analyses throughout multiple sample collection periods in order to accurately diagnose BVDV [35]. Therefore, we collected serum from the calf again 35 days after the initial sample collection. The Ct value (35.2: GE copies 3) in the serum at the third sample collection period showed an even higher increase than the one observed at the second sample collection period. In addition, no BVDV was isolated from the sample. Moreover, the titer against BVDV2 that was measured by the virus neutralization test showed seroconversion compared at the initial and second sample collection periods. Consequently, these data suggest that the calf was an AI animal that presented similarly to a PI animal. Additionally, our RT-qPCR data showed the virus loads in the serum from the calf was decreased by approximately 1/240 from the initial to the second sample collection period. This finding suggests the possibility that it might be useful to monitor GE copies with RT-qPCR in the serum upon the diagnosis of an AI animal.

Calves that are under one month of age do not have a fully functional adaptive immune system [35,36,37]. Therefore, it is important to give provide enough passive immunity via colostrum in order to protect newborn calves from pathogen infection [38]. Previous studies have reported that the half-life of BVDV antibodies that have been introduced to calves through colostrum was approximately 23 days [39]. Four AI calves were less than approximately 1 month of age when BVDV was initially detected. In addition, it is suspected that these four calves did not receive sufficient levels of BVDVs antibodies in the colostrum that they were given at the beginning of the lives, as they were shown to be low in the antibody titers that are effective against BVDVs (see Figure 2). These facts suggest that one of causative factors of the abnormal AI animal that occurred at the farm A is related to the inefficient passive immunity that the cow receives via colostrum from the dams.

In general, the PI calf is the most important reservoir of BVDV due to the shed viruses that are present in various secretions that an animal is exposed to over the course of its life [40,41]. On farm A, the abnormal AI calf (no. A1) and PI calf (no. A3) had almost the same birth date. This fact suggests the possibility that the AI calf might have been exposed to more BVDV2 shedding for a longer time because it was in close contact with the PI calf just after birth. In contrast, another AI calf (no. A2) showed subclinical signs because this calf was already two weeks old when it first came into contact with the PI calf. Furthermore, the 5′-UTR and E2 genomic sequences of the BVDVs that were detected from the three calves (nos. A1–A3) from farm A were 100% identical. Taken together, these facts suggest that the PI calf (no. A3) was an infectious source on farm A. On farm B, two PI calves (nos. B3 and B4) had already been born before the two AI calves (nos. B1 and B2) were, but they were kept at a farther distance away. Additionally, the 5′-UTR and E2 genomic sequences of the BVDVs that were found in these two calves (nos. B1 and B2) could not be determined because their genomes were absent or were only present in low quantities. Therefore, the two AI calves might not have demonstrated BVDV infection because of their low level of exposure to the viruses. However, our analysis suggests that two PI calves (nos. B3 and B4) were an infectious source on farm B.

The most important point to consider when trying to control BVDV infection is to find PI animals early and to eliminate them from the herd [20,42]. This study demonstrates that further sample collection in addition to the initial and second sample collection periods is effective for the diagnosis of AI and PI with BVDV. A previous study recommended conducting two tests 30 days apart [20,43]. In contrast, other previous studies have reported that the RNA of BVDVs can be detected in AI animals from 85 days to 95 days post-infection [25,44]. Other than, the previous studies have suggested that AI neonates and young calves with BVDV might cause a prolongation of viremia because of their inefficient immune response or because their immune response is inhibited because of immune complexes of the virus with the antibodies that are obtained through colostrum intake [45]. These facts suggest that an additional interval may be necessary to accurately diagnose AI; however, this additional interval means that the time needed to make a BVDV diagnosis is too long. In this study, the results of one calf at the second sample collection period could not deny the possibility of PI animal. However, three other AI calves were easily able to be distinguished from the PI animals with two tests within 21 days of each other. Hence, it is recommended to conduct the second test 21 days after the initial sample collection period, which would be appropriate in most diagnostic cases. In cases where it is difficult to make a diagnosis based on two tests, it is better to conduct further tests using samples collected 14–21 days after the second sample collection period.

In conclusion, our analyses for seven calves via multiple tests identified four AI calves and three PI calves who were infected with BVDV2. One AI calf could not be excluded as a possible PI after retesting 21 days after the initial sample collection period based on the OIE Terrestrial manual. However, the calf that was unable to be distinguished as being AI or PI after a third test was conducted 35 days after the initial sample collection period. Our data suggest that the prolongation of AI presenting similarly to PI might be the result of the three following factors: the immature and delayed adaptive immune response of a newborn calf, passive immunity from colostrum, and long-term virus exposure from another PI calf. The data presented herein provide useful information that can be used to help distinguish AI from PI. Further information should be accumulated through multiple analyses of more field cases focusing on BVDV outbreaks in order to contribute to better criteria for a more accurate diagnoses of BVDV in the future.

## Figures and Tables

**Table 1:** Summary of Ct values of real-time RT-PCR, results of virus isolation, S-N values of antigen detection ELISA, titers of neutralizing antibody, and genotypes of BVDV in four acutely infected calves and three persistently infected calves.

Farm	Calf No.	Clinical Signs	Age (Day)	Detection Period	Collection Date	RT-qPCR ^a^		Virus Isolation ^c^	AgELISA (S-N Value)	Neutralizing Antibody Titer ^d^		Geno Type ^e^	Status
						Ct Value ^a^	Copy per 1 µL of Serum ^b^			BVDV1	BVDV2		
A	A1	No. Died: 11 November 2017	7	1st	28 September 2017	19.3	69,000	ncp	3.656	32	neg	2	AI
			28	2nd	19 October 2017	28.1	280	ncp	3.489	16	neg		
			42	3rd	2 November 2017	35.2	3	−	1.030	4	4		
	A2	No.	21	1st	28 September 2017	28.0	290	−	0.744	neg	neg	2	AI
			42	2nd	19 October 2017	−	−	−	0.009	32	512		
	A3	Diarrhea Died: 11 October 2017	8	1st	28 September 2017	24.9	2200	ncp	3.214	neg	neg	2	PI
			21	2nd	11 October 2017	19.2	72,000	ncp, cp	3.276	neg	neg		
B	B1	No.	15	1st	4 January 2018	−	−	−	0.333	neg	32	NT	AI
			37	2nd	26 January 2018	−	−	−	0.001	32	1024		
	B2	No.	32	1st	4 January 2018	36.2	1	−	0.959	neg	neg	2	AI
			54	2nd	26 January 2018	−	−	−	−0.002	4	512		
	B3	No. Culled: 6 February 2018	84	1st	4 January 2018	25.6	1310	ncp	3.698	neg	neg	2	PI
			106	2nd	26 January 2018	25.7	1280	ncp	3.699	neg	neg		
	B4	No. Culled: 9 February 2018	116	1st	10 January 2018	24.6	2570	ncp	3.698	neg	neg	2	PI
			137	2nd	31 January 2018	25.0	1940	ncp	3.702	neg	neg		

^a^ Ct value ≦ 31, presumptive BVDV PI; 31 < Ct value < 38, positive for BVDV based on the criteria described in the instruction of the RT-PCR kit (The VetMAX-Gold BVDV PI Detection Kit); −, below detection limit (Ct value 40<). ^b^ Virus genomic equivalent (GE) copies of each sample were estimated based on the standard curves using serial dilutions of standard BVDV RNA (10,000 copies/μL) by RT-qPCR. ^c^ cp, cytopathic type; ncp, non-cytopathic-type; −, no virus isolation. ^d^ neg, means negative (antibody titers of <2). ^e^ NT, not tested.

## Data Availability

Not applicable.

## Supplementary Materials

The following are available online at https://www.mdpi.com/article/10.3390/v13122431/s1, Table S1: List of primers and probes used in BVDV genotyping by multiplex real-time RT-PCR, Table S2: List of primers used in phylogenetic analyses for 5′-untranslated region and E2 region from Pestiviruses.
