# Supplementary material for: An Importance of Long-Term Clinical Analysis to Accurately Diagnose Calves Persistently and Acutely Infected by Bovine Viral Diarrhea Virus 2
Source: Viruses. 2021 Dec 3;13(12):2431. doi: 10.3390/v13122431 (PMC8705094; doi:10.3390/v13122431)
Supplement: Supplementary file 1 [file viruses-13-02431-s001.zip › viruses-1449400-supplementary.pdf]

Supplementary Table S1. List of primers and probes used in BVDV genotyping by multiplex real-time RT-PCR.

Target virus	Function	Sequence 5'-3'
Pestiviruses	Forward primer	GATGCCATGTGGACGAGGGC
	Reverse primer	CATGTGCCATGTACAGCAGAG
Pestivirus A (BVDV1)	probe	FAM-CAATACAGTGGGCCTCTGCAGCA-QSY
Pestivirus B (BVDV2)	probe	VIC-GTGGCGTTATGGACACAGCCTG-QSY
Pestivirus H	probe	ABY-ATCAGGCTGTACTCCCAAAG-QSY

Supplementary Table S2. List of primers used in phylogenetic analyses for 5′-untranslated region and E2 region from Pestiviruses.

Target region	Function	Sequence 5'-3'
5′-untranslated region	Forward primer	ATGCCCWTAGTAGGACTAGCA
	Reverse primer	TCAACTCCATGTGCCATGTAC
E2 region	Forward primer	ACTTTGAATTTGGACTYTGCC
	Reverse primer	TCCAGGTCAAACCARTATTG
